# Changes of the Cytoplasmic Proteome in Response to Alcoholic Hepatotoxicity in Rats

**DOI:** 10.3390/ijms160818664

**Published:** 2015-08-10

**Authors:** Dong Hwan Kim, Eun-Mi Lee, Sun-Hee Do, Da-Hee Jeong, Kyu-Shik Jeong

**Affiliations:** 1 College of Interdisciplinary & Creative Studies, Konyang University, Nonsan 320-711, Korea; E-Mail: dhkim@konyang.ac.kr; 2 College of Veterinary Medicine, Kyungpook National University, Daegu 702-701, Korea; E-Mails: nikeun@hanmail.net (E.-M.L.); daheej@kotra.or.kr (D.-H.J.); 3 Stem Cell Therapeutic Research Institute, Kyungpook National University, Daegu 702-701, Korea; 4 College of Veterinary Medicine, Konkuk University, Seoul 143-701, Korea; E-Mail: shdo@konkuk.ac.kr

**Keywords:** alcohol, hepatotoxicity, proteomics, cytoplasm

## Abstract

Proteomic analyses have already been used in a number of hepatological studies and provide important information. However, few reports have focused on changes in the cytoplasmic proteome. The present study therefore aimed to evaluate changes in cytoplasmic proteome of rats in response to alcoholic hepatotoxicity. Rats were fed a Liber-DeCarli liquid diet containing ethanol for four weeks. Cytoplasmic proteins except mitochondrial proteins from the livers of these animals were investigated using two-dimensional gel electrophoresis and mass spectrometry. Alcohol induced a decrease in body weight gain and an increase in alanine transaminase (ALT), cholesterol, and phospholipid levels. Histopathological observations revealed hepatic damage characterized by necrosis and fatty change in alcohol-treated group at week 2, which continues until week 4. Our proteomic analysis revealed that 25 proteins were differentially expressed in the ethanol-fed group. Of these, 12 cytoplasmic proteins are being reported for the first time. Taken together, our results provide further insights into the disease mechanism and therapeutic information of alcoholic liver disease.

## 1. Introduction

Alcohol abuse is the leading cause of morbidity and mortality worldwide. Although alcohol has cytotoxic effects on numerous tissues, the liver is one of the most common targets. The stages of alcoholic liver disease (ALD) comprise steatosis, steatohepatitis, and fibrosis/cirrhosis [1]. The pathogenesis of ALD has been increasingly delineated over the years by a number of studies. The main causes of ALD were found to be malnutrition, redox changes by oxidation of ethanol, microsomal (especially CYP2E1) induction, formation of protein adducts from acetaldehyde and RNS/ROS formation. These result in antibody production, enzyme inactivation, decreased DNA repair, impaired utilization of oxygen, glutathione depletion, and increased collagen and ROS/RNS synthesis [2,3,4,5,6,7,8].

Relatively little, however, is known about the actual mechanisms underlying the development and progression of ALD. ALD is essentially a multifactorial disease and has been shown to be associated with changes in protein regulation that cover a wide spectrum of organic functions. Proteomics allows the simultaneous assessment of the expression, up-regulation and down regulation, of a large number of proteins, providing an unprecedented amount of information [9]. Such data can then be used to explore underlying pathophysiological mechanisms.

This approach has already been used in a number of hepatological studies. Some investigators have focused on proteomic analyses of ALD using mitochondrial, plasma membrane, and liver whole protein [10,11,12,13,14]. Although the cytoplasm plays an important role in ALD, few reports have focused on changes in cytoplasmic protein expression. Therefore, profiling cytoplasmic protein expression in ALD will provide further insights into the underlying disease mechanism and open new avenues for the discovery of novel therapeutic targets. We adopted a proteomics-based approach to analyze protein expression in the cytoplasmic but not mitochondrial fraction from the livers of rats fed a chronic oral liquid ethanol diet.

## 2. Results

### 2.1. Body Weight and Serum Biochemical Analyses

The ethanol-fed group showed lower weight gains during the experimental period than the pair-fed control group. A statistically significant decrease in body weights was observed at weeks 3 and 4 (Table 1).

Serum biochemical analyses showed that alcohol induced a significant increase in alanine transaminase (ALT), cholesterol, and phospholipid levels at 2 and 4 weeks after treatment. Our results indicated that liver injury and fat accumulation in liver cells, which appeared to be more severe at 2 weeks (Table 2). However, alkaline phosphatase (ALP) and triglyceride were not affected by chronic ethanol intake.

**Table 1:** Body weight changes after chronic ethanol consumption for four weeks.

Week	Control (mg)	Ethanol (mg)
0	104 ± 2.3	102 ± 1.4
1	124 ± 10.8	117 ± 5.7
2	137 ± 8.7	127 ± 6.6
3	166 ± 8.3	156 ± 5.7 *
4	198 ± 15.2	181 ± 5.2 *

Values represent mean ± S.D.; *, Significantly different from the control, *p* < 0.05.

**Table 2:** Serum biochemical marker levels in pair-fed rats for four weeks.

Group	ALT (IU/L)	Cholesterol (mg/dL)	HDL (mg/dL)	LDL (mg/dL)	Phospholipid (mg/dL)	Triglyceride (mg/dL)
Control (2 weeks)	36.6 ± 6.31	77.4 ± 12.5	30.4 ± 3.78	15.6 ± 5.94	142.6 ± 12.76	78.0 ± 46.50
Control (4 weeks)	35.0 ± 5.72	58.0 ± 2.94	26.0 ± 2.16	6.5 ± 1.29	102.5 ± 8.66	94.2 ± 25.75
Ethanol (2 weeks)	153.2 ± 42.38 **	114 ± 10.02 **	45.0 ± 3.74 **	21.8 ± 4.44	212.0 ± 19.34 **	37.5 ± 1.29
Ethanol (4 weeks)	81.2 ± 33.53 *	98.4 ± 22.39 *	41.8 ± 11.19 *	15.4 ± 4.39 **	170.8 ± 43.13 *	52.6 ± 33.16

Values represent mean ± S.D. (*n* = 5); *, *p* < 0.05, **, *p* < 0.01 compared to the pair-fed controls.

### 2.2. Histopathology and Immunohistochemistry (IHC)

**Figure 1:**
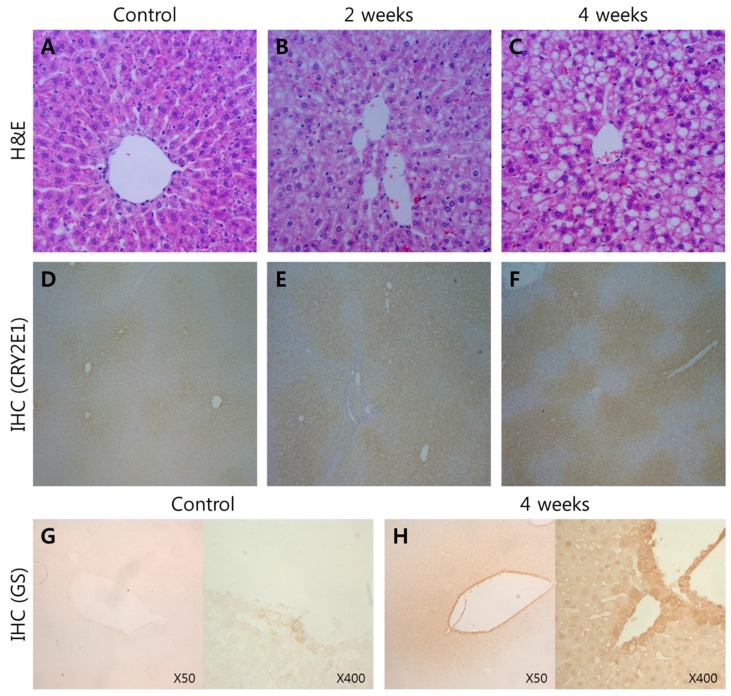
Histopathological and immunohistochemical changes in ethanol fed rat liver. (**A**) Normal control diet-fed rat liver; (**B**) Two weeks after an ethanol diet was fed to the rats. Hepatic fatty change and necrosis were visible in the cytoplasm of hepatocytes in the centrilobular areas of ethanol-fed rats; (**C**) Four weeks after the ethanol diet was fed to the rats. Hepatic fatty change and necrosis were more severe than that observed in **B**, and prominent fatty changes were observed in hepatocytes (H&E staining, ×132); (**D**) Normal control diet fed rat liver. CYP 2E1 immunoreactivity was relatively weak; (**E**) Two weeks after the rats were fed an ethanol. CYP 2E1 expression increased in pericentral hepatocytes at two weeks compared to **D**; (**F**) Four weeks after the rats were fed an ethanol. Expression of CYP 2E1 was stronger than that observed in **E** (×66); (**G**) Normal control diet fed rat liver. GS expression was week; and (**H**) Four weeks after the rats were fed an ethanol. GS strongly expressed in perivenous hepatocytes. (×50 and ×400).

Hepatic fatty change and necrosis were observed in ethanol-fed rats. At 2 weeks, lipid droplets were visible in the cytoplasm of hepatocytes in the centrilobular areas of the experimental group (Figure 1A,B). Moreover, prominent fatty changes were observed in the hepatocytes at week 4 (Figure 1C).

Immunohistochemistry showed that ethanol-inducible CYP2E1 was present in liver sections from all animals. In the control livers, CYP2E1 immunoreactivity was found to be normal (Figure 1D). However, CYP2E1 expression increased in pericentral hepatocytes of ethanol-fed rats from week 2 to week 4. Thereby indicating successful ethanol feeding, which causes hepatic injuries such as necrosis and fatty changes as well as the induction of microsomal CYP2E1 isozyme, which was a quality control in this experiment (Figure 1E,F). The control livers showed weak glutamine synthetase (GS) immunoreactivity (Figure 1G). However, GS expression was prominent and increased in perivenous hepatocytes of ethanol-fed rats from week 4 (Figure 1H).

### 2.3. Proteomic Analysis

**Figure 2:**
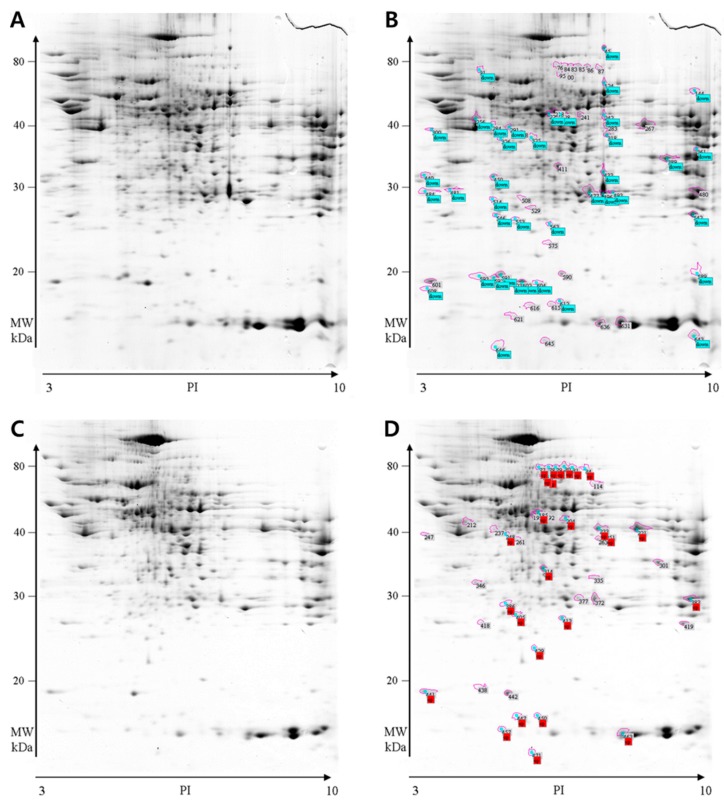
Two-dimensional electrophoretic gel images of cytoplasmic proteins. Spots indicated by spot number showed statistically significant differences in expression between the pair-fed and ethanol-fed group (*n* = 5, *p* < 0.05). (**A**,**B**) pair-fed rat liver; and (**C**,**D**) ethanol-fed rat liver; down (blue in **B**), down-regulated protein by ethanol consumption; up (red in **D**), up-regulated protein by ethanol consumption.

Using the ImageMaster™ 2D Platinum ver. 5.0 software (Amersham Biosciences, Uppsala, Sweden), around 500 to 650 protein spots were detected from each gel and 2219 individual protein spots were generated from five pairs of control and ethanol-fed rats. The abundance ratio of each spot was determined by the % volume in the gel. Image analysis revealed that 66 individual protein spots were differentially expressed in response to chronic ethanol feeding. Figure 2A,B show the SDS-PAGE images of cytoplasmic proteins in pair fed rats and Figure 2C,D show cytoplasmic proteins in ethanol fed rats. The circles indicate proteins that were significantly up- or down-regulated compared to the other groups. Of these 66 spots, 40 were down-regulated and 26 were up-regulated as a result of chronic ethanol consumption. All protein spots that showed differences in response to ethanol feeding were excised and subjected to trypsin digestion, followed by protein identification by MALDI-TOF MS and peptide mass profiling. Of these 66 protein spots, 17 down-regulated spots and 16 up-regulated spots were identified by Swiss Prot. 2005.01.06 database and are listed in Table 3 and Table 4. Of these, 14 down-regulated and 11 up-regulated proteins were unique proteins.

As shown in Table 3, the down-regulated proteins were NADH dehydrogenase (ubiquinone), transketolase (TK), peroxisomal bifunctional enzyme (PBE), 40S ribosomal protein SA (p40), sorbitol dehydrogenase (SD), sideroflexin 1 (Sfxn1), thiosulfate sulfurtransferase (rhodanese), carbonic anhydrase III (CA-III), 3-hydroxyanthranilate 3,4-dioxygenase (3-HAO), α-tocopherol transfer protein (α-TTP), protein kinase C inhibitor protein-1 (KCIP-1), peroxiredoxin 1 (Pxr1), 15.5 kDa fatty acid binding protein (FABP) and superoxide dismutase [Cu-Zn] (Cu/Zn-SOD). The up-regulated proteins (Table 4) were protein-arginine deiminase type II (PADII), transferrin, long-chain acyl-CoA synthetase 2 (LACS2), GS, argininosuccinate synthase (AS), cell division control protein 2 homolog (CDC2), cytochrome P450 (CYP), plasma retinol-binding protein precursor (PRBP), cytochrome b5, transthyretin precursor (TBPA) and hemoglobin beta chain (β-globin).

**Table 3:** Down-regulated proteins in liver cytoplasmic fractions following chronic ethanol consumption: identification of proteins from 2D IEF/SDS-PAGE gels.

Spot No. ^a^	Protein Name	*M*w (Da)/pI	MOWSE Score	Accession #	Control Mean %vol.	Ethanol Mean %vol.	Mean Fold ^b^	*p*-Value ^c^
91	NADH dehydrogenase (ubiquinone)	79,413/5.6	3.08 × 10^5^	Q66HF1	0.0460	0.0000	0.00	0.030
124	Transketolase (TK)	67,644/7.2	5.11 × 10^4^	P50137	0.0705	0.0459	0.65	0.032
144	Peroxisomal bifunctional enzyme (PBE)	78,659/9.3	251	P07896	0.0363	0.0143	0.39	0.048
300	40S ribosomal protein SA (p40)	32,824/4.8	729	P38983	0.0832	0.0406	0.49	0.028
318	Sorbitol dehydrogenase	42,835/6.8	9459	P27867	0.1499	0.0766	0.51	0.002
361	Sideroflexin1 (Tricarboxylate carrier protein, Sfx1)	35,547/9.2	401	Q63965	0.0550	0.0000	0.00	0.012
389	Thiosulfate sulfurtransferase (Rhodanese)	33,177/7.8	1537	P24329	0.4404	0.3192	0.72	0.037
433	Carbonic anhydrase III (CAIII)	29,432/6.9	4.56 × 10^5^	P14141	0.1277	0.0484	0.38	0.049
450	3-Hydroxyanthranilate 3,4-dioxygenase (3-HAO)	32,582/5.6	1.78 × 10^8^	P46953	0.1542	0.0968	0.63	0.001
477	α-Tocopherol transfer protein (α-TTP)	31,846/6.5	119	P41034	0.4700	0.1147	0.24	0.001
484	Protein kinase C inhibitor protein-1 (KCIP-1)	27,771/4.7	488	P63101M	0.0493	0.0000	0.00	0.046
493	Carbonic anhydrase III (CAIII)	29,432/6.9	69.3	P14141	0.0926	0.0000	0.00	0.047
496	Carbonic anhydrase III (CAIII)	29,432/6.9	1.07 × 10^7^	P14141	1.0801	0.2330	0.22	0.002
542	Peroxiredoxin 1 (Pxr1)	22,110/8.3	3.47 × 10^5^	Q63716	0.3207	0.1982	0.62	0.045
591	15.5 kDa fatty acid binding protein (FABP)	20,737/5.8	1.09 × 10^4^	P02761	0.1007	0.0040	0.04	0.030
602	Superoxide dismutase [Cu-Zn] (Cu/Zn-SOD)	15,912/5.9	2164	P07632	0.3729	0.2699	0.72	0.021
603	Superoxide dismutase [Cu-Zn] (Cu/Zn-SOD)	15,912/5.9	1879	P07632	0.0279	0.0021	0.08	0.041

^a^ Spot No. in Figure 1A; ^b^ average the fold change observed from five pairs of control and ethanol-fed rats; ^c^ two-tailed paired Student’s *t*-test on normalized protein spot densities obtained using ImageMaster 5.0 (Amersham Biosciences).

**Table 4:** Up-regulated proteins in liver cytoplasmic fractions following chronic ethanol consumption: identification of proteins from 2D IEF/SDS-PAGE gels.

Spot No. ^a^	Protein Name	*M*w (Da)/pI	MOWSE Score	Accession #	Control Mean %vol.	Ethanol Mean %vol.	Mean Fold ^b^	*p*-Value ^c^
77	Protein-arginine deiminase type II (PADII)	75,357/5.3	2552	P20717	0.0238	0.0879	3.69	0.036
78	Serotransferrin precursor (Transferrin)	76,365/6.9	175	P12346	0.0264	0.0726	2.75	0.001
79	Serotransferrin precursor (Transferrin)	76,365/6.9	182	P12346	0.0408	0.0914	2.24	0.000
80	Serotransferrin precursor (Transferrin)	76,365/6.9	6461	P12346	0.0710	0.1399	1.97	0.000
81	Serotransferrin precursor (Transferrin)	76,365/6.9	4.20 × 10^5^	P12346	0.0775	0.1290	1.66	0.009
84	Serotransferrin precursor (Transferrin)	76,365/6.9	705	P12346	0.0073	0.0459	6.27	0.048
91	Long-chain acyl-CoA synthetase 2 (LACS2)	78,179/6.6	5.53 × 10^5^	P18163	0.0146	0.0545	3.74	0.039
204	Glutamine synthetase (GS)	42,268/6.6	256	P09606	0.0535	0.1165	2.18	0.138
221	Argininosuccinate synthase	46,497/7.6	2.71 × 10^4^	P09034	0.7412	1.0884	1.47	0.050
233	Argininosuccinate synthase	46,497/7.6	6.03 × 10^4^	P09034	0.2361	0.3910	1.66	0.004
383	Cell division control protein 2 homolog (CDC2)	34,135/8.4	1015	P39951	0.4508	0.8771	1.95	0.015
413	Cytochrome P450	25,939/5.6	134	Q64678	0.0530	0.1032	1.95	0.027
429	Plasma retinol-binding protein precursor (PRBP)	23,220/5.7	795	P04916	0.0147	0.0251	1.71	0.049
441	Cytochrome b5	15,355/4.9	2849	gi11560046	0.2409	0.4508	1.87	0.027
447	Transthyretin precursor (TBPA)	15,720/5.8	1631	P02767	0.0263	0.0608	2.32	0.017
463	Hemoglobin beta chain (β-globin)	15,980/7.9	5.81 × 10^5^	P02091	0.6404	1.0725	1.67	0.044

^a^ Spot No. in Figure 1B; ^b^ average the fold-change observed from five pairs of control and ethanol-fed animals; ^c^ two-tailed paired Student’s *t*-test on the normalized protein spot densities obtained using ImageMaster 5.0 (Amersham Biosciences).

### 2.4. Immunoblotting Analysis

**Figure 3:**
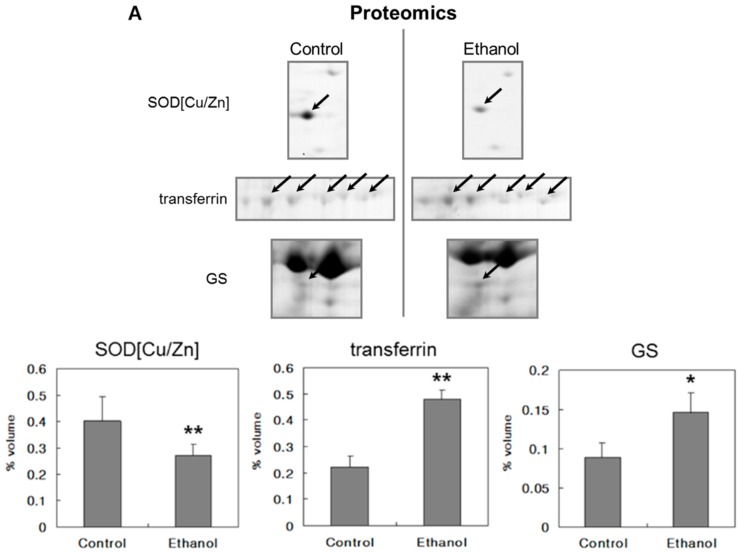

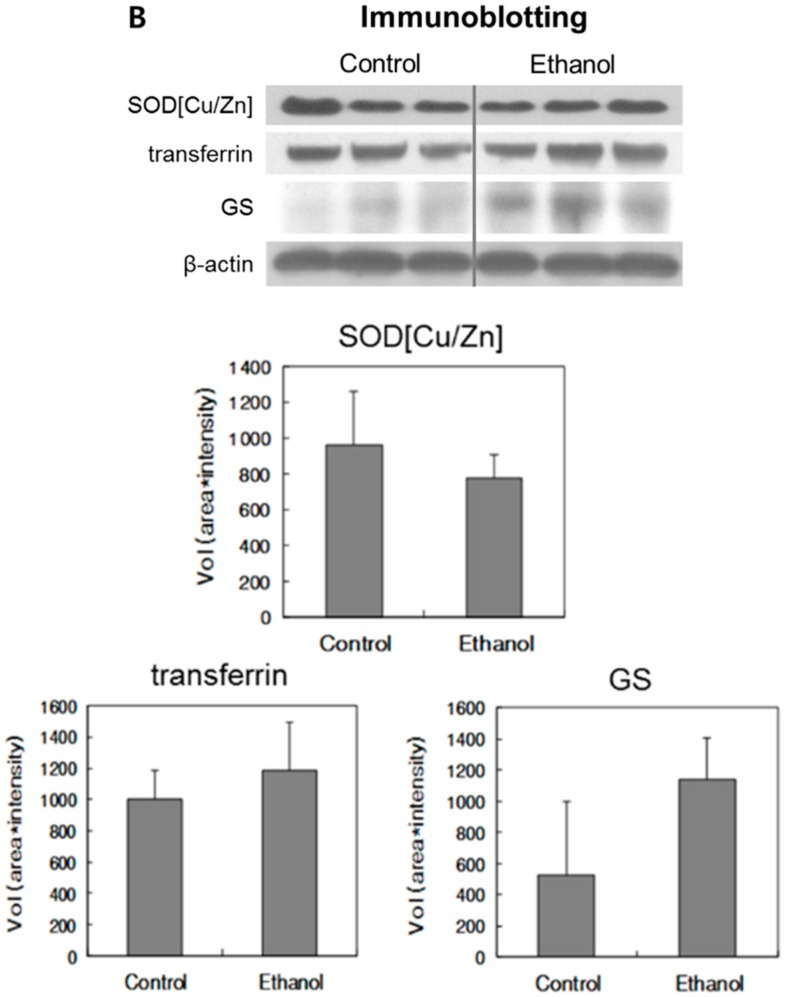
Protein spots and immunoblots of Cu/Zn-SOD, transferrin, and GS in control and ethanol-fed groups. (**A**) Cu/Zn-SOD decreased significantly in ethanol-fed rat liver compared with the pair-fed control. Comparing the Cu/Zn-SOD enzyme expression by spot volume using ImageMaster II, Cu/Zn-SOD in ethanol-fed rat liver was 0.68-fold that of control rat liver in proteomics (*n* = 5). Transferrin and GS were significantly increased in ethanol-fed group. Transferrin and GS in ethanol-fed group were 2.1-f and 2.18-fold that of the control group, respectively (*n* = 5); and (**B**) In the immunoblotting analysis (*n* = 3), the expression of Cu/Zn-SOD, transferrin, and GS in the ethanol-fed group were 0.81-, 1.2-, and 2.15-fold that of the control group, respectively. β-actin was used as a control in immunoblot. Data are shown as the mean ± SEM. (* *p* < 0.05, ** *p* < 0.01).

CYP2E1 was increased significantly in ethanol-fed rat liver compared with pair-fed control. This result correlated well with our immunohistochemistry results. Although CYP2E1 is a microsomal enzyme, the increase in CYP2E1 was not successfully detected in cytoplasmic proteome. The immunoblot of Cu/Zn-SOD showed a decrease of Cu/Zn-SOD in the ethanol-fed group (Figure 3). Futhermore, these results correlated with proteomic analysis. Cu/Zn-SOD in ethanol-fed group was 0.81-fold that of the control group as shown by immunoblotting and 0.68-fold by proteomic analysis (Table 5). Transferrin and GS increased in ethanol-fed group (Figure 3).

**Table 5:** Comparison of protein expression in the immunoblot between control and ethanol-fed groups. The volumes in immunoblot were calculated by ImageMaster II software (*n* = 3).

Protein	Mean Volume in Control Group	Mean Volume in Ethanol Group	Mean Fold in Immunoblot	Mean Fold in Proteomics ^a^
Cu/Zn-SOD	959.88	778.53	0.81	0.68
Transferrin	999.96	1191.26	1.19	2.14
GS	530.19	1141.75	2.15	2.18

^a^ Data from Figure 1A,B.

## 3. Discussion

Ethanol-fed rats body weight was significantly less than their pair-fed counterparts during the last two weeks. These findings were in accordance with those of previous studies [15,16]. A decrease in body weight gain is due to the decrease in nutritional absorption by direct impairment of the GI tract and/or metabolic disturbance of nutrition absorption by ethanol. Serum biochemical analysis revealed that alcohol induced a significant increase in ALT, cholesterol, and phospholipid levels at 2 and 4 weeks after treatment. These results were characterized by liver injury and fat accumulation in liver cells, which was more severe at 2 weeks. In general, the Lieber-DeCarli diet model produces fatty liver and metabolic tolerance; however, lesions beyond steatosis are rare [17]. Apte *et al.* reported that liver injury declined at weeks 4 and 5 and the increase in cell division was shown at week 5 in the Lieber-DeCarli diet model [18].

Histopathological evaluation revealed significant steatosis, which progressed to panlobular steatosis in the ethanol-fed rats. Both microvesicular and macrovesicular steatosis were present. Fatty infiltration was evident as early as week 2 and progressed to week 4. In addition to steatosis, significant apoptosis was also detected by week 2 of ethanol feeding, which increased further by week 4. The discrepancy in the severity in biochemistry and histopathology could be explained by histopathological and biochemical changes. This discrepancy was also shown in the other experiment, where the histopathological severity declined by week 5 of ethanol ingestion [17]. CYP2E1, a well-known microsomal protein that markedly increases after alcohol consumption, was used in immunohistochemistry and immunoblotting analysis as a quality control, was significantly increased.

**Figure 4:**
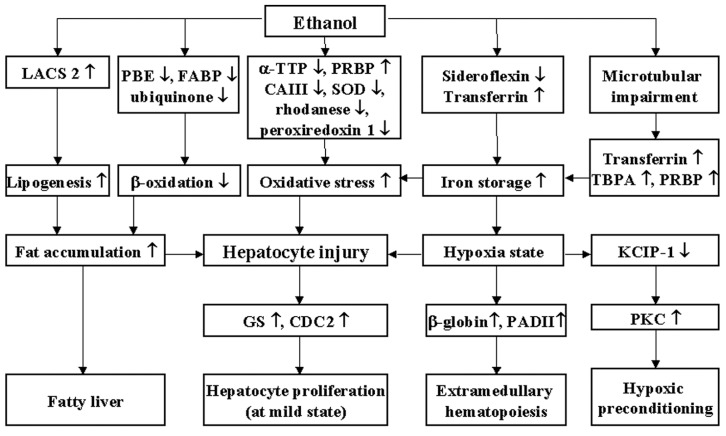
The illustration of proposed changes in cytoplasmic proteins and pathophysiology in the livers of ethanol-fed rats. “↑” and “↓” indicates the up- and down-regulation by chronic ethanol consumption, respectively. Chronic ethanol ingestion induced up-regulation of LACS2 that was followed by increase of lipogenesis in hepatocytes. Down-regulation of PBE, FABP, and ubiquinone by ethanol induced a decrease of β-oxidation. The increase of lipogenesis and decrease of β-oxidation led to fat accumulation and fatty liver. Chronic ethanol consumption also induced increase in PRBP and decrease in α-TTP, CAIII, Cu/Zn-SOD, rhodanese, and peroxiredoxin 1, which may cause of oxidative stress. Microtubular impairment in the cell membrane by ethanol induced up-regulation of transferrin, TBPA, and PRBP. These changes, in addition to the down-regulation of sideroflexin 1 and up-regulation of transferrin, can lead to the increase in iron storage in hepatocytes. Excessive iron can create a hypoxic state in liver cells and induce oxidative stress. All of these changes attack liver cells chronically and induce hepatocyte injury. Mildly injured liver tissue can produce GS and CDC2 for restoration and hepatocyte proliferation. However, a hypoxic state may induce extramedullary hematopoiesis by increase of β-globin and PADII and hypoxic preconditioning state by down-regulation of KCIP-1, which can lead to PKC secretion.

We applied proteomics to study the changes of expression of cytoplasmic proteins in rats fed a liquid diet containing ethanol. In the proteomic analysis, 17 down-regulated spots (14 proteins) and 16 up-regulated spots (11 proteins) were identified by Swiss Prot. 2005.01.06 database. The differential expression of these proteins and proposed pathophysiological mechanisms were illustrated in Figure 4.

Among these fourteen down-regulated proteins, six proteins such as ubiquinone [19], CAIII [20,21,22], 3-HAO [14], Pxr1 [23,24,25], FABP [19,26,27], and Cu/Zn-SOD [28] were previously reported to be down-regulated by chronic alcohol ingestion.

Ubiquinone destroys radicals that are normally produced within cells and are toxic to the biological system. Alcohol metabolism results in a shift in the cytoplasmic [NAD+]/[NADH] ratio to reduction, which in turn causes a direct inhibition of β-oxidation and enhanced triacylglycerol formation via the [glycerol-3-phosphate]/[dihydroxyacetone phosphate] ratio. Eaton *et al.* [19] reported that chronic alcohol ingestion leads to ubiquinone down-regulation. Decreased amounts of ubiquinone could lead to fat accumulation and increase in free radical damage. Therefore, TK is a key enzyme in the pentose-phosphate pathway and glucose metabolism. TK is a thiamine-dependent enzyme and thiamine deficiency is a common feature in patients with chronic alcoholism, and has been considered to mainly the result from alcoholism, regardless of the underlying liver disease. Erythrocyte TK activity was reported to decrease ALD [29]; however, the relationship of liver TK and alcohol were not reported previously. The down-regulation of TK may impair the energy metabolism and lead to thiamine deficiency.

PBE is a β-oxidation-related enzyme located in the peroxisome. Decreased PBE reduces fatty acid metabolism and cause hepatopathy. The same event was shown in hepatocellular carcinoma [30]. Sfxn1 might be involved in transport of a component required for iron utilization into or out of the mitochondria [31]. Sfxn1 down regulation might impair iron utilization and energy production using oxygen.

Rhodanese, which catalyzed the transfer of a sulfane sulfur atom from an anionic donor to a thiophilic acceptor via an enzymic persulfide (ESS-) intermediate [32], was originally believed to have a direct role in the conversion of inorganic cyanide to the less toxic thiocyanate anion. Nandi *et al.* [33] suggested that one of the functions of rhodanese is the detoxication of intra-mitochondrial oxygen free radicals. Down-regulation of rhodanese in this experiment might increase oxidative stress with other down-regulated proteins such as α-TTP, CAIII, Cu/Zn-SOD, and Pxr1.

CAIII is a major participant in the liver’s response to oxidative stress. In an oxidizing environment, CAIII undergoes oxidative modification, decreasing the phosphatase activity that is characteristic to its native form. Several reports revealed that CAIII decreased in rats after ethanol feeding [20,21,22]. In contrast, CAIII protein expression in the micropig liver increases as a result of ethanol administration.

α-TTP binds with α-tocopherol and enhances its transfer between separate membranes. Therefore, α-TTP down regulation decreases α-tocopherol (antioxidant) transfer and induces fatty liver and cirrhosis [34]. Wizmann *et al.* reported that protein kinase C levels increased at an ischemic or hypoxic preconditioning state [24].

KCIP-1 downregulation might result in a concomitant increase in protein kinase C levels and lead to hypoxic preconditioning of hepatocytes. And Pxr1 functions to reduce peroxides with reducing equivalents provided through the thioredoxin system but not from glutaredoxin. Pxr1 may play an important role in eliminating peroxides generated during alcohol metabolism. Therefore, decreased Pxr1 expression may induce oxidative stress and cause liver injury.

In a study of protein expression in the nucleus accumbens and hippocampus of inbred alcohol-preferring and -non-preferring rats, peroxiredoxin showed 1.5- and 1.2-fold abundance in alcohol-preferring rats compared with that of alcohol-non-preferring rats [24]. The induction of hepatic extramitochondrial pathways of fatty acid oxidation via PPAR serve to provide hepatocytes with alternative means for the fatty acid catabolism under conditions of marked increased fatty acid flux and fatty acid “overload”. Liver fatty acid binding protein is clearly integral to this response and may act to reduce the toxicity of long-chain unesterified fatty acids by binding them in the cytoplasmic compartment and facilitating their intracellular diffusion and utilization. ALD is associated with a state of hepatic fatty acid overload. Nanji *et al.* [27] examined the effect of ethanol and different types of dietary fat on the expression of mRNA for liver fatty acid binding protein, peroxisome proliferator-activated receptor-α, and peroxisomal fatty acyl-CoA oxidase. They concluded that adaptive gene regulation of FACO and l-FABP by PPAR is impaired in ethanol-fed rats.

Cu/Zn-SOD is a major cytoplasmic enzyme, which destroys radicals that are normally produced within the cells and are toxic to biological systems. Cu/Zn-SOD is a ubiquitous chain-breaking antioxidant and is found in all aerobic organisms. It is a widely distributed metalloprotein and plays an important protective role against ROS-induced oxidative damage. SOD converts superoxide radicals to H_2_O_2_ and the hydrogen peroxide thus formed is degraded by catalase and glutathione peroxidase. Catalase is present in all major organs of animals and humans and is especially concentrated in the liver and erythrocytes. Administering ethanol (6.32 g/kg body weight) to mice for 45 days resulted in decreased SOD and catalase activity in the liver of mice receiving alcohol [28]. Reduced SOD and catalase activity in the presence of ethanol may cause the accumulation of superoxide (O_2_^−^), H_2_O_2_, or the products of their decomposition. Loss of catalase and SOD activity results in oxygen intolerance and triggers a number of deleterious reactions. It has therefore been proposed that the contribution of catalase might be enhanced if significant amounts of H_2_O_2_ become available through β-oxidation of fatty acids in peroxisomes [35]. Decreased Cu/Zn-SOD was confirmed by immunoblotting. Cu/Zn-SOD in ethanol-fed group was 0.81- and 0.68-fold that of the control group as shown by immunoblotting and proteomic analysis, respectively. Mechanisms or functions of other down-regulated cytoplasmic proteins such as P40 and sorbitol dehydrogenase by alcohol ingestion remain to be elucidated.

Of the 11 up-regulated proteins, seven cytoplasmic proteins such as LACS2, GS, argininosuccinate synthase, CDC2, cytochrome P450, PRBP, and cytochrome b5 were previously reported to be up-regulated as a result of chronic alcohol ingestion. Other proteins up-regulated by chronic alcohol consumption such as transferrin, TBPA, hemoglobin beta-2 chain, and PADII were reported for the first time in this study.

Of the up-regulated proteins, transferrin, PRBP, and TBPA are secreted from the hepatocytic Golgi apparatus. Increases in the levels of these proteins were secondary to acetaldehyde-induced impairment of microtubule-mediated protein secretion associated with engorgement of the Golgi apparatus [15,36]. Increased transferrin and TBPA expression and decreased Sfxn1 expression in hepatic cytoplasm increased iron storage in hepatocytes and decreased iron utilization. This might result in increased oxidative stress by the Fenton reaction in the cytoplasm and hypoxic state in cells and organs.

Increased transferrin expression was confirmed by immunoblotting. Transferrin in the ethanol-fed group was 1.19-fold higher than that in the control group; however, it was found to be 2.14-fold higher by proteomic analysis. Rats exposed to hypercapnic/hypoxic conditions showed significantly increased extramedullary hematopoiesis in the liver and hemoglobin β-2 chain levels in serum [37].

PADII is the primary enzyme responsible for conversion of protein-bound arginine to citrulline in the central nervous system (CNS) and was also increased in human astrocytes by prolonged hypoxic exposure. Chronic alcohol consumption might produce hypoxic conditions in the liver as explained above and might induce increases of β-globin and PADII.

CDC2 plays a key role in the control of the eukaryotic cell cycle proliferation. Apte *et al.* [20] reported that NF-κB DNA and CDC2 proteins increased significantly in the ethanol-treated rats, which corresponded with enhanced hepatic proliferation. It was also suggested that promotion of GS expression in a new pericentral hepatocyte of CCl_4_-treated mice and proliferative events play an important role in up-regulation of GS [38]. Considering these results, mild impairment of hepatocyte may increase GS and CDC2 in liver cells and provoke hepatocyte proliferation. In the immunoblot, GS in the ethanol-fed group were 2.15-fold higher than that of the control group. This result matched well with our proteomic analysis results of GS in the ethanol-fed group, which was found to be 2.18-fold higher than that in the control group.

LACS2 functions to activate of long-chain fatty acids for both cellular lipid synthesis, and degradation via β-oxidation. Long-term ethanol administration increased LACS gene expression 1.7-fold, suggesting that alcohol stimulates lipogenesis in the liver [39]. These results are concordant with our data and could explain the increase of fat accumulation in hepatocytes. Bunout [40] reported that the global effect of alcohol is to induce protein loss and to increase nitrogen excretion. As argininosuccinate synthase plays a major role in nitrogen metabolism, an increase in the expression of these enzymes can be explained by nitrogen excretion. The exact mechanism, however, should be further evaluated.

## 4. Experimental Section

### 4.1. Animals

Male Wistar rats used in this study were housed individually in an automatically controlled environment (23 ± 3 °C, 50% ± 10% relative humidity) with a 12-h light/dark cycle. During the 4 weeks of experimentation, these animals were fed a Liber-DeCarli liquid diet containing ethanol or an isocalorically balanced diet with maltose dextrin, for the pair-fed controls. Fifteen animals per group were used in this study. Experimental diet sources were purchased from Dytes Inc. (Philadelphia, PA, USA). This was an improved version of the original Liber-DeCarli diet, administered as a liquid suspension according to a previously reported protocol by Matsuda *et al.* [15]. All experimental procedures were performed in accordance with the NIH guidelines for the care and use of laboratory animals.

### 4.2. Body Weight Changes and Serum Biochemical Analyses

The body weights of rats were recorded once a week. ALT, ALP, total cholesterol, triglyceride, HDL, LDL, and phospholipids were measured by standard enzymatic procedures (Konelab 20, Thermo Clinical Labsystems, Vantaa, Finland).

### 4.3. Histopathology and Immunohistochemistry

Liver tissues were fixed in 10% neutral buffered formalin, processed routinely and embedded in paraffin wax. Sections were cut to a thickness of 4 μm and stained with hematoxylin-eosin.

For immunohistochemical analysis, tissue sections were immunostained with the primary antibody; the primary antibodies used were CYP2E1 and GS at a dilution of 1:200 (Merk Millipore, Billerica, MA, USA; BD biosciences, San Jose, CA, USA). The antigen-antibody complex was visualized by the streptavidin-biotin method using a Histostatin-plus bulk kit (Zymed Laboratories Inc., San Francisco, CA, USA).

### 4.4. Proteomic Analysis

#### 4.4.1. 2D PAGE Sample Preparation

For 2D PAGE sample preparation, liver tissues were suspended in sample buffer (40 mM Tris, 9 M urea, 4% CHAPS, 1 mM EDTA, 10 mM dithioerythritol) containing a protease inhibitor cocktail tablet (Roche, Germany). The mixture was centrifuged at 5000 rpm (about 2000× *g*) for 10 min at 4 °C to remove solid tissue and cell debris. The supernatant as the total homogenate was centrifuged at 12000 rpm (about 12,000× *g*) for 10 min at 4 °C to separate out the mitochondrial proteins. Trichloroacetic acid (TCA) 50% solution (*w*/*v*) was added to the supernatant to a final concentration of 10% (*w*/*v*) and the solution was allowed to stand on ice for 30 min. The protein precipitate was collected by centrifugation at 12,000 rpm (about 12,000× *g*) for 15 min at 4 °C. The precipitate was dried by using Speedvac. The dry pellet was dissolved in the sample buffer without the protease inhibitor cocktail tablet and allowed to stand for 1 h at room temperature. After centrifugation at 15,000 rpm (about 19,000× *g*) for 60 min at 15 °C, the supernatant was used as the 2D gel electrophoresis sample for the soluble cytoplasmic protein fraction. Protein concentration of 2DE samples was estimated using a commercial 2D Quant Kit (Amersham Biosciences).

#### 4.4.2. First Dimension (IEF)

First dimensional isoelectric focusing (IEF) experiment was carried out on 18 cm, pH 3–10 immobilized pH gradient (IPG) strips (Amersham Biosciences) at 20 °C with a maximum current setting of 50 μA/strip on an IPGphor electrophoretic unit (Amersham Biosciences). The strips were rehydrated overnight in DeStreak Rehydration solution (Amersham Biosciences) containing 0.5% IPG buffer (Amersham Biosciences). After rehydration, protein samples were loaded onto the anodic end of the IPG strip using the loading cup. IEF was performed according to the following steps; 500 V for 1 h, 1000 V for 1 h, 1000–8000 V for 2000 Vh and 8000 V for 32,000 Vh.

#### 4.4.3. Two-Dimensional Gel Electrophoresis

After isoelectric focusing, the strips were equilibrated. The first equilibration solution contained 30% (*w*/*v*) glycerol, 6 M urea, 2% (*w*/*v*) sodium dodecyl sulfate, 50 mM Tris-HCl, pH 8.8, 65 mM dithiothreitol, and a trace of bromophenol blue. The second equilibration was performed with the first equilibration solution, except that DTT was replaced by 260 mM iodoacetamide. Second-dimensional SDS-polyacrylamide gel electrophoresis was then performed. Separation for second-dimension was performed on 1.0 mm thick 12% polyacrylamide gels at 10 °C. The gels were run at 40 mA per gel in an Ettan DALT apparatus (Amersham Biosciences), accommodating six gels. After protein fixation for 12 h in 40% methanol and 10% acetic acid, the gels were stained with Coomassie blue (Bio Basic, Amherst NY USA) for 1 h. Electronic images of the gels were obtained by PowerLook 1120 (UMAX Systems GmbH, Willich, Germany), recorded and quantified using the ImageMaster 2D software (Amersham Biosciences) [41].

#### 4.4.4. MALDI-TOF MS

For protein identification, spots were picked from the gel and de-stained with 30% methanol. Enough 200 mM ammonium bicarbonate was added to the gel piece, and mixed. The gel pieces were shrunk by dehydration in 100% acetonitrile and then the pieces were then dried. Enzymatic digestion was performed by the addition of 0.0125 μg/μL sequence-grade modified trypsin (Promega, Madison, WI, USA) in 50 mM ammonium bicarbonate and 5 mM calcium chloride, and incubated at 37 °C for 16 h. Extracted digestion mixtures were dried, and then suspended in 0.1% trifluoroacetic acid (TFA). The extracted sample was dispensed onto the MALDI sample plate with matrix solution, consisting of a saturated solution of 40 mg/mL α-cyano-4-hydroxy-cinnamic acid in 50% acetonitrile, containing 0.1% TFA. Samples were dried under ambient conditions. Mass spectra were obtained using Voyager-DE STR MALDI-TOF mass spectrometer (Thermo Fisher Scientific, Waltham, MA, USA) with delayed extraction and reflectron. Spectra were calibrated upon acquisition using the ProteoMass Peptide MALDI-MS Calibration Kit, MS-CAL2 (Sigma, St. Louis, MO, USA) with angiotensin II (1046.5423), P14R (1533.8582) and ACTH fragment 18–39 (2465.1989) as external calibration and angiotensin I (1296.48) as an internal standard. The automatically identified proteins were checked individually and only rat proteins with pI and molecular mass values close to the theoretical considered (a deviation of about 20% was allowed). The database searches were carried out with the MS-Fit (Available online: http://prospector.ucsf.edu).

### 4.5. Immunoblotting

For the immunoblot, snap-frozen liver tissues of three rats per group were homogenized in RIPA buffer and protease inhibitor cocktail tablets (Roche, Basel, Switzerland). The lysate was centrifuged at 3000 rpm (about 750× *g*) for 10 min at 4 °C to remove solid tissue and debris. Subsequently, supernatant was centrifuged at 13,000 rpm (about 14,000× *g*) for 20 min at 4 °C to obtain soluble cytoplasmic proteins. For immunoblotting, proteins were electro-transferred onto a PVDF membrane (Schleicher & Schuell, Dassel, Germany). CYP2E1 (Leica Microsystems, Wetzlar, Germany), Cu/Zn-SOD (Stressgen, Victoria, BC, Canada), transferrin (Dakocytomation, Glostrup, Denmark) and GS (BD biosciences, San Jose, CA, USA) were detected using monoclonal mouse antibody (1:500). Specific binding was detected using the Super Signal West Dura Extended Duration Substrate (Thermo Fisher Scientific).

### 4.6. Statistical Analysis

All numerical data were expressed as mean ± standard deviation. Statistical analysis of the data was performed using the Microsoft Excel program. Student’s *t*-test was conducted for comparison of the control and ethanol-fed group. *p* < 0.05 or *p* < 0.01 was considered statistically significant.

## 5. Conclusions

This study is the first systematic analysis of the cytoplasmic proteome (excluding the mitochondrial proteome) in response to ethanol ingestion. Fourteen down-regulated and 11 up-regulated cytoplasmic proteins in response to ethanol feeding were identified by proteomic analysis and confirmed by immunohistochemistry and immunoblotting. Our results provide further insights into mechanisms underlying ALD, and clinically, the discovery of novel proteins can be provide new therapeutic information for ALD treatment.

## References

[1] Ramaiah S., Rivera C., Arteel G. (2004). Early-Phase Alcoholic Liver Disease: An update on animal models, pathology, and pathogenesis. Int. J. Toxicol..

[2] Levene A.P., Goldin R.D. (2012). The Epidemiology, Pathogenesis and histopathology of fatty liver disease. Histopathology.

[3] Leung T.M., Nieto N. (2013). CYP2E1 and oxidant stress in alcoholic and non-alcoholic fatty liver disease. J. Hepatol..

[4] Lee Y.A., Wallace M.C., Friedman S.L. (2015). Pathobiology of liver fibrosis: A translational success story. Gut.

[5] Singal A.K., Charlton M.R. (2012). Nutrition in alcoholic liver disease. Clin. Liver Dis..

[6] Arteel G.E. (2003). Oxidants and antioxidants in alcohol-induced liver disease. Gastroenterology.

[7] Brunt E.M., Tiniakos D.G. (2002). Pathology of steatohepatitis. Best Pract. Res. Clin. Gastroenterol..

[8] Ishak K.G., Zimmerman H.J., Ray M.B. (1991). Alcoholic liver disease: Pathologic, pathogenetic and clinical aspects. Alcohol. Clin. Exp. Res..

[9] Fountoulakis M. (2001). Proteomics: Current Technologies and applications in neurological disorders and toxicology. Amino Acids.

[10] Andringa K.K., King A.L., Eccleston H.B., Mantena S.K., Landar A., Jhala N.C., Dickinson D.A., Squadrito G.L., Bailey S.M. (2010). Analysis of the liver mitochondrial proteome in response to ethanol and *S*-adenosylmethionine treatments: Novel molecular targets of disease and hepatoprotection. Am. J. Physiol. Gastrointest. Liver Physiol..

[11] Bailey S.M., Andringa K.K., Landar A., Darley-Usmar V.M. (2008). Proteomic approaches to identify and characterize alterations to the mitochondrial proteome in alcoholic liver disease. Methods Mol. Biol..

[12] Venkatraman A., Landar A., Davis A.J., Chamlee L., Sanderson T., Kim H., Page G., Pompilius M., Ballinger S., Darley-Usmar V. (2004). Modification of the mitochondrial proteome in response to the stress of ethanol-dependent hepatotoxicity. J. Biol. Chem..

[13] Zhang L., Jia X., Feng Y., Peng X., Zhang Z., Zhou W., Zhang Z., Ma F., Liu X., Zheng Y. (2011). Plasma membrane proteome analysis of the early effect of alcohol on liver: Implications for alcoholic liver disease. Acta Biochim. Biophys. Sin..

[14] Newton B.W., Russell W.K., Russell D.H., Ramaiah S.K., Jayaraman A. (2009). Liver proteome analysis in a rodent model of alcoholic steatosis. J. Proteome Res..

[15] Matsuda Y., Takada A., Takase S., Sato H. (1991). Accumulation of glycoprotein in the golgi apparatus of hepatocytes in alcoholic liver injuries. Am. J. Gastroenterol..

[16] Aleynik M.K., Lieber C.S. (2001). Dilinoleoylphosphatidylcholine decreases ethanol-induced cytochrome P4502E1. Biochem. Biophys. Res. Commun..

[17] Gao Y., Shan Y.Q., Pan M.X., Wang Y., Tang L.J., Li H., Zhang Z. (2004). Protein kinase C-dependent activation of P44/42 mitogen-activated protein kinase and heat shock protein 70 in signal transduction during hepatocyte ischemic preconditioning. World J. Gastroenterol..

[18] Apte U.M., McRee R., Ramaiah S.K. (2004). hepatocyte proliferation is the possible mechanism for the transient decrease in liver injury during steatosis stage of alcoholic liver disease. Toxicol. Pathol..

[19] Eaton S., Record C.O., Bartlett K. (1997). Multiple biochemical effects in the pathogenesis of alcoholic fatty liver. Eur. J. Clin. Investig..

[20] Parkkila S., Halsted C.H., Villanueva J.A., Vaananen H.K., Niemela O. (1999). Expression of testosterone-dependent enzyme, carbonic anhydrase III, and oxidative stress in experimental alcoholic liver disease. Dig. Dis. Sci..

[21] Yamada M., Satoh M., Seimiya M., Sogawa K., Itoga S., Tomonaga T., Nomura F. (2013). Combined proteomic analysis of liver tissue and serum in chronically alcohol-fed rats. Alcohol. Clin. Exp. Res..

[22] Kharbanda K.K., Vigneswara V., McVicker B.L., Newlaczyl A.U., Bailey K., Tuma D., Ray D.E., Carter W.G. (2009). Proteomics reveal a concerted upregulation of methionine metabolic pathway enzymes, and downregulation of carbonic anhydrase-III, in betaine supplemented ethanol-fed rats. Biochem. Biophys. Res. Commun..

[23] Fernando H., Wiktorowicz J.E., Soman K.V., Kaphalia B.S., Khan M.F., Shakeel Ansari G.A. (2013). Liver proteomics in progressive alcoholic steatosis. Toxicol. Appl. Pharmacol..

[24] Witzmann F.A., Li J., Strother W.N., McBride W.J., Hunter L., Crabb D.W., Lumeng L., Li T.K. (2003). Innate differences in protein expression in the nucleus accumbens and hippocampus of inbred alcohol-preferring and -nonpreferring rats. Proteomics.

[25] Cesaratto L., Vascotto C., D’Ambrosio C., Scaloni A., Baccarani U., Paron I., Damante G., Calligaris S., Quadrifoglio F., Tiribelli C. (2005). Overoxidation of peroxiredoxins as an immediate and sensitive marker of oxidative stress in HepG2 cells and its application to the redox effects induced by ischemia/reperfusion in human liver. Free Radic. Res..

[26] Smathers R.L., Galligan J.J., Stewart B.J., Petersen D.R. (2011). Overview of lipid peroxidation products and hepatic protein modification in alcoholic liver disease. Chem. Biol. Interact..

[27] Nanji A.A., Dannenberg A.J., Jokelainen K., Bass N.M. (2004). Alcoholic liver injury in the rat is associated with reduced expression of peroxisome proliferator-α (PPARα)-regulated genes and is Ameliorated by PPARα activation. J. Pharmacol. Exp. Ther..

[28] Balasubramaniyan V., Kalaivani Sailaja J., Nalini N. (2003). Role of leptin on alcohol-induced oxidative stress in Swiss mice. Pharmacol. Res..

[29] Levy S., Herve C., Delacoux E., Erlinger S. (2002). Thiamine deficiency in hepatitis C virus and alcohol-related liver diseases. Dig. Dis. Sci..

[30] Suto K., Kajihara-Kano H., Yokoyama Y., Hayakari M., Kimura J., Kumano T., Takahata T., Kudo H., Tsuchida S. (1999). Decreased expression of the peroxisomal bifunctional enzyme and carbonyl reductase in human hepatocellular carcinomas. J. Cancer Res. Clin. Oncol..

[31] Zheng H., Ji C., Zou X., Wu M., Jin Z., Yin G., Li J., Feng C., Cheng H., Gu S. (2003). Molecular cloning and characterization of a novel human putative transmembrane protein homologous to mouse sideroflexin associated with sideroblastic anemia. DNA Seq..

[32] Finazzi Agro A., Federici G., Giovagnoli C., Cannella C., Cavallini D. (1972). Effect of sulfur bindng on rhodanese fluorescence. Eur. J. Biochem..

[33] Nandi D.L., Horowitz P.M., Westley J. (2000). Rhodanese as a thioredoxin oxidase. Int. J. Biochem. Cell Biol..

[34] Bell H., Bjorneboe A., Eidsvoll B., Norum K.R., Raknerud N., Try K., Thomassen Y., Drevon C.A. (1992). Reduced concentration of hepatic alpha-tocopherol in patients with alcoholic liver cirrhosis. Alcohol Alcohol..

[35] Bradford B.U., Enomoto N., Ikejima K., Rose M.L., Bojes H.K., Forman D.T., Thurman R.G. (1999). Peroxisomes are involved in the swift increase in alcohol metabolism. J. Pharmacol. Exp. Ther..

[36] Matsuda Y., Takada A., Takase S., Yasuhara M. (1991). Effects of ethanol on the secretion of hepatic secretory protein in rat alcoholic liver injury. Alcohol.

[37] Kouno A., Inoue H., Bajanowski T., Maeno Y., Iwasa M., Nakayama M., Nishi K., Brinkmann B., Matoba R. (2000). Development of Haemoglobin subtypes and extramedullary haematopoiesis in young rats. Effects of hypercapnic and hypoxic environment. Int. J. Leg. Med..

[38] Ueberham E., Arendt E., Starke M., Bittner R., Gebhardt R. (2004). Reduction and expansion of the glutamine synthetase expressing zone in livers from tetracycline controlled TGF-β1 transgenic mice and multiple starved mice. J. Hepatol..

[39] Sugimoto T., Yamashita S., Ishigami M., Sakai N., Hirano K., Tahara M., Matsumoto K., Nakamura T., Matsuzawa Y. (2002). Decreased microsomal triglyceride transfer protein activity contributes to initiation of alcoholic liver steatosis in rats. J. Hepatol..

[40] Bunout D. (1999). Nutritional and Metabolic Effects of Alcoholism: Their relationship with alcoholic liver disease. Nutrition.

[41] Gorg A., Obermaier C., Boguth G., Harder A., Scheibe B., Wildgruber R., Weiss W. (2000). The current state of two-dimensional electrophoresis with immobilized pH gradients. Electrophoresis.

